# Molecular and physiological consequences of faulty eukaryotic ribonucleotide excision repair

**DOI:** 10.15252/embj.2019102309

**Published:** 2019-12-12

**Authors:** Vanessa Kellner, Brian Luke

**Affiliations:** ^1^ Institute of Molecular Biology (IMB) Mainz Germany; ^2^ Institute of Developmental Biology and Neurobiology (IDN) Johannes Gutenberg Universität Mainz Germany; ^3^ Present address: Department of Biology New York University New York NY USA

**Keywords:** DNA repair, ribonucleotide excision repair, RNA–DNA hybrid, RNase H2, topoisomerase 1, DNA Replication, Repair & Recombination

## Abstract

The duplication of the eukaryotic genome is an intricate process that has to be tightly safe‐guarded. One of the most frequently occurring errors during DNA synthesis is the mis‐insertion of a ribonucleotide instead of a deoxyribonucleotide. Ribonucleotide excision repair (RER) is initiated by RNase H2 and results in error‐free removal of such mis‐incorporated ribonucleotides. If left unrepaired, DNA‐embedded ribonucleotides result in a variety of alterations within chromosomal DNA, which ultimately lead to genome instability. Here, we review how genomic ribonucleotides lead to chromosomal aberrations and discuss how the tight regulation of RER timing may be important for preventing unwanted DNA damage. We describe the structural impact of unrepaired ribonucleotides on DNA and chromatin and comment on the potential consequences for cellular fitness. In the context of the molecular mechanisms associated with faulty RER, we have placed an emphasis on how and why increased levels of genomic ribonucleotides are associated with severe autoimmune syndromes, neuropathology, and cancer. In addition, we discuss therapeutic directions that could be followed for pathologies associated with defective removal of ribonucleotides from double‐stranded DNA.

## Graphical abstract

Kellner and Luke review how RNase H2 or topoisomerase enzymes remove misincorporated ribonucleotides from genomic DNA, and the relevance of such mechanisms for both malignant and autoimmune diseases such as Aicardi–Goutières syndrome.

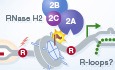

**Glossary**
AGS: Aicardi–Goutières syndrome
AOA1: Ataxia with oculomotor apraxia 1
dNTP: deoxyribonucleotide triphosphate
DSB: double‐strand break
GCR: gross chromosomal rearrangements
HDR: homology‐directed repair
HR: homologous recombination
INF: interferon
LOH: loss of heterozygosity
MMR: mismatch repair
NER: nucleotide excision repair
NHEJ: nonhomologous end‐joining
PARP: (poly‐ADP)ribose polymerase
PIP‐box: PCNA‐interacting motif
RER: ribonucleotide excision repair
RNAPII: RNA polymerase II
RNase H: ribonuclease H
rNMP: ribonucleoside monophosphate
rNTP: ribonucleotide triphosphate
SLE: systemic lupus erythematosus
SSB: single‐strand break
ssDNA: single‐stranded DNA
Top1cc: topoisomerase 1 cleavage complex
Top1: topoisomerase 1

## Introduction

It is of critical importance that the duplication of human genomes is a tightly regulated and safe‐guarded process. The insertion of erroneous bases by replicative DNA polymerases can potentially manifest into heritable mutations with pathological consequences. Considerable emphasis has been placed on understanding the causes, consequences, and repair of faulty base incorporation, which leads to DNA mismatches. In some instances, the replicative polymerases still get it wrong despite the correct base being inserted and a Watson–Crick basepair being formed; this can occur when the inappropriate sugar moiety attached to the base is selected.

The replication of DNA utilizes deoxyribonucleotide triphosphates (dNTPs) to faithfully duplicate the genome. The synthesis of RNA via transcription, on the other hand, employs ribonucleotide triphosphates (rNTP), where the 2′‐carbon atom of the ribose sugar is hydroxylated. Intracellular concentrations of rNTPs highly exceed those of dNTPs (between 30‐ and 200‐fold in budding yeast depending on the base) (Nick McElhinny *et al*, 2010b), thereby increasing the likelihood that rNTPs are mistakenly used instead of dNTPs, despite the presence of the correct base attached. The three replicative DNA polymerases (Pol α, Pol ε, and Pol δ) harbor a highly conserved tyrosine residue adjacent to the active polymerization site that acts as “steric gate” to limit rNTP incorporation during DNA replication, by excluding the hydroxy group of the ribose (Brown & Suo, 2011). rNTP exclusion via the steric gate is however not flawless, and *in vitro* studies using endogenous concentrations of rNTPs and dNTPs suggest that in the *Saccharomyces cerevisiae* genome, approximately 13,000 ribonucleotides become incorporated into newly replicated DNA within a single round of replication (Nick McElhinny *et al*, 2010b). Similar frequencies of rNTP incorporation have been confirmed *in vivo*, resulting in an incorporation rate of approximately one rNMP per 6,500 bases (Lujan *et al*, 2013). In addition to rNTP mis‐incorporation, Pol α/Primase synthesizes short RNA primers to initiate Okazaki fragments that transiently make up ~ 5% of the nascent lagging strand (Zheng & Shen, 2011). Accordingly, in human cells the number of incorporated ribonucleotides per cell cycle is estimated to reach more than one million (Clausen *et al*, 2013). This makes ribonucleotides the most frequently incorporated non‐canonical nucleotide in duplex DNA, exceeding the combined total number of all abasic, oxidized, and otherwise modified nucleotides (Caldecott, 2014).

Once the rNTP is incorporated into the context of DNA, it exists as a ribonucleoside monophosphate (rNMP). The presence of rNMPs in a DNA template directly affects the processivity of DNA polymerization during semi‐conservative replication. Although budding yeast replicative polymerases can bypass a single rNMP, the *in vitro* efficiency of this bypass is strongly reduced (for Pol ε only about 66% bypass is achieved) and drops dramatically when replication has to transverse stretches of three or more consecutive rNMPs (Watt *et al*, 2011). As of yet, there is no direct *in vivo* evidence that DNA polymerases are affected by stretches of multiple rNMPs. Comparable *in vitro* observations have been made for the human replicative polymerases, Pol ε and Pol δ (Göksenin *et al*, 2012; Clausen *et al*, 2013). Therefore, the accumulation of rNMPs in genomic DNA likely induces replication stress and DNA damage signaling from yeast to human (Nick McElhinny *et al*, 2010a; Hiller *et al*, 2012; Lazzaro *et al*, 2012; Williams *et al*, 2013; Pizzi *et al*, 2015; Zimmermann *et al*, 2018) (discussed below). Thus, to preserve genome stability, rNMPs have to be efficiently removed from the genome. A dedicated repair pathway, referred to as ribonucleotide excision repair (RER), employs specialized enzymes to eliminate rNMPs.

## Error‐free and error‐prone removal of ribonucleotides

DNA polymerases have a built‐in proofreading mechanism whereby faulty base incorporation is corrected through exonuclease‐mediated removal of the incorrect base (Burgers & Kunkel, 2017). Recognition and excision of rNMPs by DNA polymerases is only one‐third as effective as their proofreading of incorrect dNMP base pairing and in the case of Pol ε has been found to most likely not significantly contribute to rNMP removal (Shcherbakova *et al*, 2003; Williams *et al*, 2012). Instead, RNase H enzymes have the capacity to cleave DNA at sites of rNMP incorporation and thus to initiate rNMP removal. RNase H is conserved in prokaryotes, but we will not cover bacterial RNase H enzymes here and instead refer interested readers to a study by Kochiwa *et al* (2007) for an overview on them. The eukaryotic RNase H family consists of the monomeric RNase H1 enzymes and the trimeric RNase H2 enzymes, both of which eliminate RNA‐DNA hybrid structures occurring throughout the genome (Cerritelli & Crouch, 2009). While the accidentally incorporated rNMPs are found both as single bases and in longer consecutive stretches, RNA–DNA hybrids can also form when single‐stranded RNA molecules anneal to a complementary DNA strand, thereby displacing the second strand of the DNA double helix. This three‐stranded structure, termed R‐loop, can have detrimental consequences on genome stability if not removed in a timely manner (Santos‐Pereira & Aguilera, 2015). RNase H1 requires at least four consecutive rNMPs to recognize an RNA–DNA hybrid structure, a situation that occurs in the context of an R‐loop. RNase H2, on the other hand, can act both on rNMP stretches such as those found in R‐loops, as well as on single and consecutive rNMPs in the context of double‐stranded DNA, making it a more versatile enzyme. Consistently, RNase H2 activity accounts for the bulk of RNase H activity in the cell (Sparks *et al*, 2012). Given the enzymatic capabilities of RNase H2, one may expect the existence of tight regulatory mechanisms in order to prevent chromosomal nicking at inappropriate times, e.g., during DNA replication.

In *S. cerevisiae*, the trimeric RNase H2 enzyme consists of the catalytic subunit Rnh201 and the accessory subunits Rnh202 and Rnh203. Human RNase H2 shows strong conservation and comprises RNASEH2A, the catalytic subunit, as well as RNASEH2B and RNASEH2C (Crow *et al*, 2006). Loss of any of its subunits renders the enzyme complex inactive (Jeong *et al*, 2004). The dual activity of RNase H2 toward R‐loops and rNMPs can be largely, but not entirely, uncoupled by the use of a separation‐of‐function allele of the catalytic Rnh201 subunit, *RNH201‐P45D‐Y219A* (or *RNH201‐RED* for ribonucleotide excision defective). This point mutation within the substrate‐interacting pocket completely abolishes activity of RNase H2 toward single rNMPs but retains approximately 40% of wildtype enzymatic activity toward longer rNMP stretches and R‐loops (Chon *et al*, 2013). Although this allele was originally constructed in yeast, it has since been recapitulated in human and mouse cells, resulting in a similar separation‐of‐function phenotype (Pizzi *et al*, 2015; Uehara *et al*, 2018; Zimmermann *et al*, 2018).

### RNase H2 promotes error‐free RER

**Figure 1:**
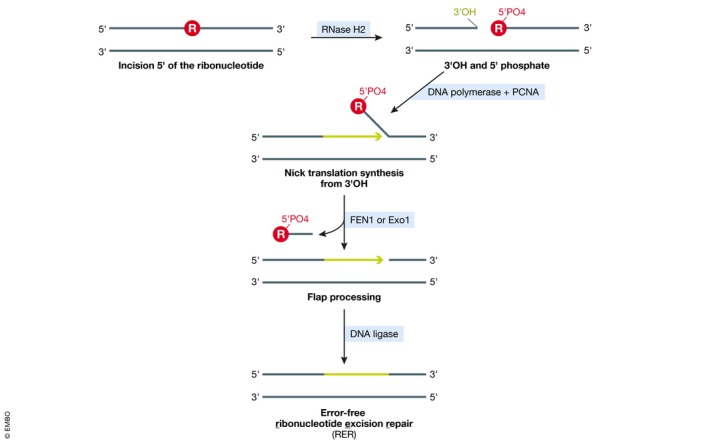
Overview of ribonucleotide excision repair (RER) RNase H2 initiates RER by incising the DNA backbone at the rNMP (R in red circle). Nick translation DNA synthesis from the newly created 3′OH followed by FEN1/Exo1‐mediated flap processing and subsequent DNA ligation can efficiently repair the incised DNA, resulting in removal of the rNMP.

RNase H2 is responsible for the primary pathway of rNMP removal from genomic DNA, i.e., error‐free RER. Upon recognizing an rNMP in the context of duplex DNA, RNase H2 incises the DNA backbone on the 5′ side of the ribonucleotide to allow its subsequent removal and repair (Eder *et al*, 1993; Rydberg & Game, 2002; Fig 1). *In vitro* reconstitution experiments have elucidated the RER mechanism in detail (Sparks *et al*, 2012): Initial incision at the DNA–RNA junction mediated by RNase H2 produces a single‐stranded DNA break flanked by a 3′ hydroxy (3′OH) group and a 5′ phosphate. Starting from the 3′OH, Pol δ or (less efficiently) Pol ε perform strand displacement DNA synthesis, thereby creating a flap structure harboring the rNMP (Fig 1). This flap is subsequently removed by flap endonuclease (yeast Rad27/human FEN1) or the exonuclease Exo1. Finally, the remaining single‐stranded nick is sealed by DNA ligase (Sparks *et al*, 2012). Crystal structures of bacterial, mouse, and human RNase H2 enzymes have allowed to further dissect their substrate recognition, binding, and hydrolysis mechanisms (Rychlik *et al*, 2010; Shaban *et al*, 2010; Figiel *et al*, 2011): RNase H2 recognizes rNMPs at the (5′)RNA–DNA(3′) junction. The 5′‐phosphate of the rNMP is positioned into the active site of the complex, while its 2′OH interacts with a glycine–arginine–glycine (GRG) motif and a conserved tyrosine residue within the catalytic subunit, thereby improving substrate selectivity (Rychlik *et al*, 2010). The catalytic step takes place in the active site consisting of four conserved carboxylates, which coordinate metal ions and water molecules to attack the phosphate bond 5′ of the rNMP (Rychlik *et al*, 2010; Shaban *et al*, 2010).

### Error‐prone rNMP removal by Top1 in the absence of RNase H2

**Figure 2:**
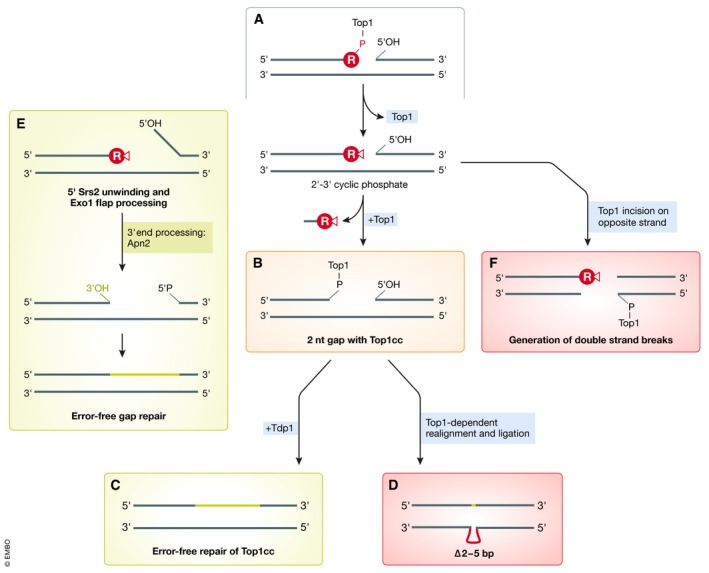
Topoisomerase 1 as backup for RER in rNMP removal from the genome (A) In the absence of functional RNase H2, Top1 can act on accumulating rNMPs. Different outcomes have been characterized in budding yeast (see text for detailed descriptions), resulting either in error‐free repair or in repair that causes mutations or potentially lethal double‐strand breaks. (B–D) Secondary Top1‐mediated incision two basepairs upstream releases an rNMP‐dNMP dinucleotide and creates a Top1‐linked gap (B) that can be processed in an error‐free manner via Tdp1 (C), or in an error‐prone manner caused by Top1 realignment and religation (D). (E) Error‐free gap repair based on subsequent activities of Srs2 helicase, Exo1 exonuclease, and Apn2 abasic endonuclease, which prevent erroneous religation. (F) Secondary Top1 incision on the opposite strand creates DNA double‐strand breaks that require repair by homologous recombination.

Prior to characterization of the RNase H2‐based RER mechanism in such intricate detail, *in vitro* work had demonstrated that topoisomerase 1 (Top1) can also process an rNMP‐containing DNA substrate (Sekiguchi & Shuman, 1997). More recently, this Top1‐dependent mechanism was also shown to remove rNMPs from DNA *in vivo* and thus to represent an important backup mechanism in RER‐defective cells lacking RNase H2 activity (Williams *et al*, 2013). In this reaction, the catalytic tyrosine residue of Top1 forms a Top1 cleavage complex (Top1cc), i.e., a covalent intermediate via transesterification at the 3′ terminal phosphate of the rNMP, in a similar manner to the reaction that Top1 initiates on pure supercoiled DNA lacking rNMPs. During the latter, canonical reaction, Top1 can readily catalyze religation of the created nick; when the nick occurs at an rNMP, however, the Top1‐phosphate bond is prone to attack from the adjacent 2′OH group of the ribose moiety, resulting in Top1 release and creation of an unligatable 2′,3′‐cyclic phosphate (Sekiguchi & Shuman, 1997; Fig 2A). The resulting nick flanked by the 2′,3′‐cyclic phosphate and a 5′OH group requires further processing before either ligation or extension is possible. Although Top1 cleavage can achieve error‐free repair of rNMPs, other more detrimental repair alternatives exist (Fig 2). In fact, Top1‐mediated processing of rNMPs greatly contributes to genome instability in the absence of RNase H2 (discussed below).

Top1‐mediated removal has been described to be specific for rNMPs incorporated by the leading‐strand DNA polymerase Pol ε, and it appears to be resolved in different ways (Williams *et al*, 2013, 2015; Cho *et al*, 2015). In one scenario, Top1 can initiate a second cut on a dNMP two basepairs upstream of the initial cut, leading to the release of an rNMP‐dNMP dinucleotide (Sparks & Burgers, 2015; Fig 2B). In this case, the covalently bound Top1cc is processed and released by tyrosyl‐DNA phosphodiesterase Tdp1, leaving behind a two‐nucleotide gap that can be repaired in an error‐free manner (Fig 2C). As an alternative to this Tdp1‐dependent pathway, and especially within tandem repeat sequences, Top1 may realign the DNA backbone and ligate the nick (Huang *et al*, 2015; Sparks & Burgers, 2015). In this scenario, ligation by Top1 leads to characteristic slippage mutations consisting of two‐ to five‐basepair deletions (∆2–5 bp) (Nick McElhinny *et al*, 2010a; Kim *et al*, 2011; Fig 2D). Processing of the initial Top1‐created 5′OH via Srs2 helicase and Exo1 nuclease can disfavor direct religation, thereby reducing the risk of acquiring those ∆2–5 bp slippage mutations (Potenski *et al*, 2014). Here, the 3′–5′ helicase activity of Srs2 unwinds the DNA from this free 5′ end, followed by flap processing via Exo1. While creation of this DNA gap reduces the likelihood of Top1‐mediated ligation following a second Top1 incision, the 3′ DNA end still has to be processed to allow extension by Pol δ. Biochemical *in vitro* data supported by genetic interaction studies indicate that the abasic endonuclease Apn2 can process the 3′‐terminal 2′,3′‐cyclic phosphate and promote Pol δ extension (Li *et al*, 2019; Fig 2E).

Yet another scenario related to yeast Top1 activity at rNMP sites has been described: Following the first incision at the rNMP, Top1 can also cut on the DNA strand opposing the rNMP to create double‐strand breaks (DSBs), which then rely on homology‐directed repair (HDR) via Rad51 and Rad52 (Huang *et al*, 2015; Fig 2F). Consistently, RNase H2 loss in human cells results in synthetic lethality with the absence of either BRCA1 or BRCA2 HDR factors, highlighting the importance of HDR under conditions of rNMP accumulation (Zimmermann *et al*, 2018).

While rNMP removal via topoisomerase had initially only been described in *S. cerevisiae*, recent work employing various RER‐defective RNase H2‐mutant human cell lines has demonstrated that also human TOP1 can recognize an unrepaired rNMP and incise to create a DNA nick with the potential to compromise genome stability (Zimmermann *et al*, 2018). Whether TOP1 activity indeed removes rNMPs from human DNA as it does in yeast remains to be tested. In particular, it will be interesting to determine how Top1 recognizes rNMPs in the DNA, i.e., is Top1‐mediated rNMP processing a regulated process, or rather an accidental byproduct of Top1 action in relieving supercoiling? Another outstanding question is whether or not Top1 contributes to RER in the presence of RNase H2, and if so to what extent. Moreover, what determines which Top1 repair pathway (nicking out the rNMP, nicking and re‐ligating or DSB generation) will be employed at an rNMP? The latter question is particularly relevant in the context of RER‐defective cells, where the different Top1‐dependent processing mechanisms will have very different outcomes in terms of preserving genome integrity.

## Alternative removal mechanisms and tolerance of rNMPs

Both yeast and mammalian cells lacking RNase H2 display signs of replication stress. In yeast mutants, the S‐phase checkpoint and the postreplicative repair pathway are constitutively activated, and accordingly, cells exhibit delayed cell cycle progression (Nick McElhinny *et al*, 2010a; Lazzaro *et al*, 2012; Williams *et al*, 2013; Zimmermann *et al*, 2018). The same defects are observed in RNase H2‐depleted human cells and cells from patients suffering from Aicardi–Goutières syndrome (AGS) associated with mutations in RNase H2 (discussed below) (Pizzi *et al*, 2015). Similarly, loss of RNase H2 in mouse cells results in altered cell cycle timing with accumulation of cells in G2/M phase, chronic activation of the DNA damage response, increase in single‐strand breaks (SSBs), and increased nuclear foci harboring the phosphorylated histone variant H2AX (γH2AX) (Hiller *et al*, 2012). In bacteria, nucleotide excision repair (NER) can serve as backup for RER (Cai *et al*, 2014), but a NER contribution to rNMP removal in yeast and human cells has been largely ruled out (Lazzaro *et al*, 2012; Lindsey‐Boltz *et al*, 2015). Mismatch repair (MMR), which is very efficient in recognizing and removing mismatched bases from dsDNA, also does not appear to contribute to rNMP removal in yeast (Lazzaro *et al*, 2012). Of note, RNase H2‐mediated incision at rNMPs has conversely been assigned a guiding role during MMR: As rNMPs are transiently inserted during DNA replication, nicks subsequently created by RNase H2 serve as a guide for strand determination and ensure that the MMR machinery specifically removes mismatches on the newly synthesized strand discriminated by the nicks (Ghodgaonkar *et al*, 2013; Lujan *et al*, 2013). Therefore, RNase H2 defects may increase the mutagenic load also in an indirect manner, by decreasing the efficiency of MMR.

Of note, the lethality of combined absence of both RNase H enzymes and Top1 in yeast (*rnh1Δ rnh201Δ top1Δ*) can be bypassed by expression of the RER‐deficient, but R‐loop processing‐proficient *RNH201‐RED* allele (Chon *et al*, 2013). This indicates that lethality in this strain is likely mediated by an accumulation of toxic R‐loops and implies that yeast cells can in principle tolerate the rNMPs that accumulate in the genome when both RER and Top1‐dependent repair are compromised. Therefore, either a yet unknown backup pathway for rNMP removal might exist, or yeast cells can survive high numbers of rNMPs in their genome despite a high load of DNA damage and replication stress. In cells that lack both RNase H1 and RNase H2, the postreplicative repair pathway has been found to be crucial (Lazzaro *et al*, 2012), but it has not been addressed to which extent this is due to either R‐loops or rNMPs, leaving the involvement of this pathway in the bypass of lethality allowed by the *RNH201‐RED* allele an interesting hypothesis to test. Another possibility to consider is that stretches of consecutive ribonucleotides, and not R‐loops, are responsible for the phenotypes observed in *rnh1Δ rnh201Δ* cells. This would also be consistent with a genetic rescue by the *RNH201‐RED* allele. Recent work has demonstrated that translesion polymerase eta (Pol η) can incorporate consecutive rNMP stretches at stalled replication forks in the presence of HU (Meroni *et al*, 2019). Consistently, the deletion of Pol η rescues the HU sensitivity of *rnh1Δ rnh201Δ* cells.

Given the importance of RER for maintaining genome integrity, it is surprising that DNA polymerases have evolved to permit rNTP usage at all. This may suggest that, pending their timely and controlled removal, rNMPs may also exert beneficial functions in certain situations (Potenski & Klein, 2014). As discussed above, MMR on the nascent leading strand is facilitated by RNase H2‐induced nicks at rNMPs (Ghodgaonkar *et al*, 2013; Lujan *et al*, 2013), and it is an intriguing possibility that other processes could be similarly affected by rNMPs incorporated by replicative DNA polymerases. Another source of rNMP incorporation with a beneficial role is in nonhomologous end‐joining (NHEJ) repair, where a critical role is played by DNA polymerase mu (Pol μ), which displays even weaker sugar selectivity than replicative DNA polymerases (Potenski & Klein, 2014). Indeed, transient incorporation of rNMPs at broken DNA ends effectively enhances their subsequent ligation (Pryor *et al*, 2018).

## RER regulation

Though many aspects of the RER reaction have been well‐described, some rather fundamental aspects of the error‐free removal of rNMPs still require further investigation. The C‐terminal region of Rnh202 (hRNASEH2B) harbors a PCNA‐interacting peptide motif (PIP‐box) suggesting replisome association, the importance of which still remains unclear. While deletion of the PIP‐box affects localization of RNase H2 to sites of PCNA‐dependent DNA replication in human cells (Bubeck *et al*, 2011), an RNase H2 complex lacking the PIP‐box (RNASEH2B‐∆PIP) still retains residual co‐localization with PCNA (Kind *et al*, 2014). In budding yeast, Rnh202‐∆PIP‐mutant cells grow indistinguishably from cells with wildtype Rnh202, suggesting that recognition of the rNMP rather than interaction with PCNA is crucial for RER to function (Chon *et al*, 2013). One possibility is that, especially in human cells, PCNA might reinforce retention of RNase H2 at sites of DNA replication or repair synthesis but is not required for its recruitment *per se*. This is reminiscent of the recruitment mechanism reported for DNA methyltransferase Dnmt1, where ablation of the PIP‐box is compensated by direct interaction between the Dnmt1‐targeting sequence and DNA (Schneider *et al*, 2013), or the dual recruitment of poly(ADP‐ribose) glycohydrolase PARG through both its substrate poly(ADP‐ribose) (PAR) and its interaction with PCNA (Mortusewicz *et al*, 2011). Another simple interpretation of these results would be that RNase H2 interacts with additional replisome components other than PCNA, which too may assist RNase H2 delivery to rNMPs. Alternatively, RNase H2 may recruit PCNA and other repair factors to rNMPs to promote RER, instead of the other way around. In any case, the significance of the PIP‐box within the RNase H2 complex requires clarification.

While *RNH201* mRNA expression peaks twice during the yeast cell cycle, during S phase and again during G2/M phase (Arudchandran *et al*, 2000), the protein accumulates progressively from G1 (where expression is very low) through to M phase (Lockhart *et al*, 2019). All other, non‐catalytic RNase H2 subunit proteins are constitutively expressed throughout the cell cycle, and the complex resides exclusively within the nuclear compartment (Reijns *et al*, 2012; Lockhart *et al*, 2019). Thus, RER could theoretically be initiated at any given time in the cell cycle. However, it is conceivable that RER may somehow be temporally regulated, or even coordinated with other DNA repair activities, in order to avoid untimely DNA nick generation and repair synthesis. Although the PIP‐box presence in RNase H2 may imply RER coupling to DNA replication, other DNA metabolism and repair processes (such as Okazaki fragment maturation and postreplicative repair) have been found to be postponable until late S/G2 phase without compromising their efficiency (Daigaku *et al*, 2010; Karras & Jentsch, 2010; Kahli *et al*, 2019). While RNase H2 did not localize to particular genomic regions in chromatin immunoprecipitations from asynchronously growing yeast cells (Zimmer & Koshland, 2016), it could be cross‐linked to telomeres in cells synchronized late in S phase, at times when the bulk of genomic DNA has been replicated (Graf *et al*, 2017). Consistently, upon fractionation of cell lysates, RNase H2 is more prominently chromatin‐associated in G2/M than in S phase, but to a lesser extent in G1 (Lockhart *et al*, 2019), suggesting that postreplicative chromatin association of RNase H2 may be a more general feature and not just restricted to telomeres. The same work further demonstrated that G2 phase‐restricted RNase H2 expression is sufficient to allow its functions both in R‐loop removal and RER; while restricting expression to S phase in fact causes defects in R‐loop processing as well as RER‐related toxicity (Lockhart *et al*, 2019). It may thus be crucial to limit the peak of RNase H2 activity to a postreplicative period, possibly to minimize generation of DNA double‐strand breaks arising from encounters of an oncoming replication fork with RNase H2‐induced nicks during S phase. Although risky, RNase H2 activity during S phase does likely still exist and may represent a tolerance pathway for dealing with rNMPs that have not been efficiently removed during the previous cell cycle, and for preventing Top1‐mediated genome instability.

**Figure 3:**
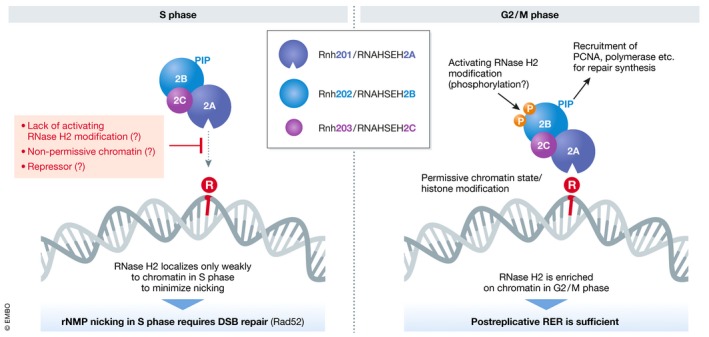
Regulation of RER through RNase H2 RNase H2 chromatin localization gradually increases throughout S phase but its activity may be kept in check to prevent creation of nicks during replication, where they would be converted into one‐ended DSBs by oncoming replication forks. Different regulatory mechanisms (in red) might account for RNase H2 inactivity during S phase. In G2/M, RNase H2 actively processes rNMPs to achieve successful RER.

Follow‐up studies to understand the mechanistic basis of RNase H2 cell cycle regulation will be an important next step. Chromatin localization could potentially be regulated by posttranslational modifications of RNase H2 or a cell cycle‐regulated RNase H2 inhibitory protein. With respect to the former, a yeast phosphoproteomics screen (Bodenmiller *et al*, 2010) yielded several phosphopeptides for Rnh202, incidentally the same RNase H2 subunit that also harbors the PIP‐box. The identification and characterization of RNase H2 posttranslational modifications could give valuable insights into the regulation of RER activity. In addition, cell cycle‐specific chromatin modifications could act as a recruitment signal for RNase H2 and need to be evaluated in this context (Fig 3).

## Unrepaired rNMPs affect genome stability

Ribonucleotides that permanently reside in the genome can have detrimental consequences on genome stability. Due to the free 2′OH group of the ribose moiety, rNMPs are highly susceptible to spontaneous hydrolysis, thus creating genotoxic single‐stranded breaks in the DNA backbone (Li & Breaker, 1999). In yeast, the absence of RNase H2 increases spontaneous mutation rates as well as gene conversion events (Huang *et al*, 2003; Ii *et al*, 2011). Moreover, rNMP‐dependent gross chromosomal rearrangements (GCRs) are observed when homologous recombination (HR)‐mediated repair is compromised (Allen‐Soltero *et al*, 2014). The most characteristic type of mutation upon loss of RER manifests in short deletions in repetitive sequences, mostly between two and five basepairs in length (Nick McElhinny *et al*, 2010a). This mutational signature is generated by Top1 incision at rNMPs followed by faulty Top1‐dependent alignment of the nicked DNA strand and religation (see above) (Kim *et al*, 2011; Williams *et al*, 2017). Increased mutation rates are similarly Top1‐dependent (Potenski *et al*, 2014). Loss of RNase H2 activity in diploid yeast cells has been shown to increase both nonallelic HR and loss‐of‐heterozygosity (LOH) events (Conover *et al*, 2015; Zimmer & Koshland, 2016; Cornelio *et al*, 2017). Although the latter study attributed LOH largely to R‐loop misregulation (Zimmer & Koshland, 2016), parallel work found LOH and nonallelic HR increased due to Top1‐dependent processing of rNMPs in RER‐defective cells (Conover *et al*, 2015). Follow‐up work employing the *RNH201‐RED* separation‐of‐function allele however concluded that both the RER and the R‐loop removal function of RNase H2 contribute to genome stability in yeast (Cornelio *et al*, 2017). Similarly, the recent findings (discussed above) that TOP1 depletion in RNase H2‐deficient human cells reduces γH2AX‐marked damage foci and PARP inhibitor‐induced apoptosis to wildtype levels and relieves the S‐phase arrest (Zimmermann *et al*, 2018) indicate that compromised RER enhances TOP1‐dependent genome instability also in human cells.

## Unrepaired rNMPs affect DNA structure

Many lines of *in vitro* evidence suggest that the presence of unrepaired rNMPs in the context of double‐stranded DNA affects the structure of the surrounding bases. Early studies analyzed short DNA molecules containing a single ribonucleotide by X‐ray diffraction (Ban *et al*, 1994; Egli *et al*, 1993; Wahl & Sundaralingam, 2000) or nuclear magnetic resonance (NMR) (Jaishree *et al*, 1993; Chou *et al,* 1991) and consistently report that the B‐form conformation usually adopted by DNA shifts either partially (i.e., locally) or completely to the A‐form typical of RNA molecules. More recent NMR work on a twelve‐basepair B‐form DNA molecule containing a single rNMP suggests that the ribose adopts an A‐form conformation, while surrounding dNMPs retain B‐conformations (Derose *et al*, 2012). These local disturbances of residues adjacent to the rNMP are consistent with atomic force microscopy (AFM)‐based studies revealing perturbed backbone elasticity in such rNMP‐containing DNA molecules (Chiu *et al*, 2014). Similar AFM analysis employing DNA molecules of up to one kilobase in length (i.e., more closely mimicking the *in vivo* context of rNMP sites) containing randomly incorporated rCTPs demonstrated shortening and increased elasticity of the DNA backbone (Meroni *et al*, 2017), suggesting that rNMPs can profoundly impact on the conformation of large DNA molecules beyond just their sites of incorporation.

Albeit difficult to prove experimentally, it is conceivable that these structural effects of rNMPs on the surrounding DNA may play an important role to enhance their recognition and subsequent removal. In the absence of functional RER, however, such structural perturbations may at the same time interfere with DNA transactions such as replication, repair, and nucleosome assembly. A plausible expectation is that DNA–protein interactions, especially those that occur in a sequence‐independent manner with proteins, would be disturbed to the greatest extent.

## Effects of unrepaired rNMPs on chromatin and chromatin‐related processes

**Figure 4:**
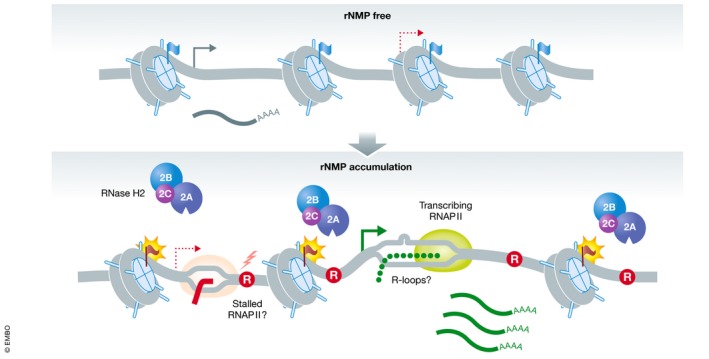
Possible chromatin alterations associated with rNMPs The accumulation or insertion of rNMPs (R in red circle) may affect the local chromatin environment by altering histone modifications on nucleosomes (flags) or even nucleosome stability and histone occupancy. These local chromatin changes may be important to allow RER by RNase H2. Such chromatin alterations may also lead to more open chromatin, increasing the expression of lowly expressed genes and thus enhancing formation of R‐loops. RNA polymerase stalling at persistent rNMPs may further impact on transcription processes.

The most common sequence‐independent DNA–protein interaction is represented by DNA wrapping around histones at the level of the nucleosome. In this regard, *in vitro* studies point toward a strong negative impact of RNA‐containing DNA on nucleosome formation (Dunn & Griffith, 1980; Hovatter & Martinson, 1987). The consequential defects in nucleosome formation and stability could result in severe consequences on cellular fitness. Less stable nucleosomes may cause altered occupation patterns, decreased nucleosome density, and consequently a less densely packed genome. Such global effects are likely to influence cellular processes that are regulated by the state of chromatin, including gene expression, chromosome segregation, and DNA repair (Fig 4). Recent molecular dynamics simulations addressing the direct effect of ribonucleotides in the context of the nucleosome have modeled a single rNMP at different positions within a DNA‐bound histone octamer (Fu *et al*, 2018). Interestingly, nucleosome formation was differentially affected by both the translational setting (distance from nucleosome midpoint) and the rotational setting (facing outwards or toward the nucleosome) of the rNMP. Outward‐facing rNMPs, independent of their translational position, adopted a C2′‐endo conformation (associated with B‐DNA) and did not affect adjacent base pairs, rendering them indistinguishable from dNMPs in terms of their overall structure. In contrast, inward‐facing rNMPs, especially at a translational position that directly interacts with the nucleosome, were mostly found in a stable C3′‐endo conformation (associated with A‐DNA) and consequently increased the width of the DNA minor grove, disrupted Watson–Crick base pairing, and induced local unwinding of the DNA duplex (Fu *et al*, 2018). Besides demonstrating a direct effect of rNMPs on DNA structure, these findings also implicate that RER efficiency might depend on the position of the rNMP within the nucleosome. An altered DNA structure may serve as a prerequisite to signal to chromatin‐remodeling enzymes that subsequently create a chromatin environment that favors RER (Fig 4). Therefore, specific chromatin marks could support recruitment of RNase H2, either in addition to or redundant with the PIP‐box of RNase H2. It is interesting to note that inward‐facing rNMPs resulted in local DNA unwinding, which would also be more permissive to R‐loop formation. Thus, the accumulation of genomic rNMPs may be tightly linked to R‐loop accumulation in this respect.

A more open global chromatin environment due to decreased nucleosome stability would most likely affect transcription. Transcriptional analysis of yeast cells lacking RNase H2 function identified a subset of 349 differentially expressed genes, with approximately one‐third of them being upregulated and two‐thirds being downregulated (Arana *et al*, 2012). Loss of RNase H2 function leads to two phenotypic consequences: an accumulation of genomic rNMPs and of R‐loops (Cerritelli & Crouch, 2009). While the exact contribution of each RNase H2 function remains undefined in this transcriptional analysis, changes in gene expression have been correlated with an increased need for DNA repair and recombination factors, cell cycle‐regulatory proteins, and the induction of a general stress response (Arana *et al*, 2012). It cannot be discriminated whether accumulation of R‐loops or genomic instability induced by unrepaired rNMPs is causal for this transcriptional response. The ability of R‐loops to induce local chromatin compaction (Castellano‐Pozo *et al*, 2013) can also not be ruled out as contributing to the transcriptional changes observed. Therefore, future investigations using the *RNH201‐RED* separation‐of‐function mutant (Chon *et al*, 2013) may allow for clearer distinction between contributions of unrepaired rNMPs and R‐loops. Moreover, including spike‐in controls in RNA‐sequencing experiments (Lovén *et al*, 2012; Chen *et al*, 2016) would allow the detection of global changes in gene expression levels, as they could be caused by a more open chromatin structure.

In line with such transcriptomics studies, another crucial aspect to be tested is whether unrepaired rNMPs may impede the progression of RNA polymerases as much as progression of replicative DNA polymerases (Watt *et al*, 2011; Göksenin *et al*, 2012; Clausen *et al*, 2013). In this case, rNMPs could physically influence RNA transcription to varying degrees, depending on whether the rNMP resides in the transcribed or the non‐transcribed strand, whether a gene is expressed at high or low levels, and where within the gene the rNMP is located. Transcription‐associated mutagenesis can indeed be linked to unrepaired ribonucleotides (Cho & Jinks‐Robertson, 2017). The characteristic two‐ to five‐basepair slippage mutations generated by Top1 at unrepaired rNMPs are elevated within actively transcribed hotspot regions (Takahashi *et al*, 2011). At low levels of transcription, those mutation events are biased toward the leading strand (Cho *et al*, 2015), which harbors the majority of rNMPs (Nick McElhinny *et al*, 2010a,b). However, high transcription levels lead to loss of this strand bias during DNA replication, with mutations accumulating preferably in the non‐transcribed strand of a reporter gene (Cho *et al*, 2015). This led to the model that RNA polymerase II (RNAPII) might physically affect the ability of Top1 to religate the nicked DNA: RNAPII might push Top1 cross‐linked on the transcribed strand toward the nick, thus supporting alignment and error‐free ligation of the DNA, but conversely would push Top1 that has incised the non‐transcribed strand away from the nick (or alternatively shield the 5′OH opposite of the Top1cc), thereby hampering ligation and favoring mutagenesis. It is important to keep in mind that ribonucleotide‐mediated mutagenesis brought about by transcription can happen in both cycling and non‐cycling cells.

## Compromised RER causes Aicardi–Goutières syndrome

RNase H2 is mutated in more than 50% of cases of the autosomal recessive genetic disorder Aicardi–Goutières syndrome (AGS) (Crow *et al*, 2006; Rice *et al*, 2007). This rare early‐onset autoinflammatory disease is reminiscent of both congenital viral infections and the autoimmune disease systemic lupus erythematosus (SLE), affecting brain and skin cells as well as the immune system (Crow & Manel, 2015). While unresolved R‐loops may contribute to disease pathology (Lim *et al*, 2015), DNA damage responses in RNase H2‐depleted human culture cells and mouse embryos, as well as in AGS patient‐derived cells, have demonstrated to be mainly caused by accumulation of rNMPs in DNA (Pizzi *et al*, 2015; Uehara *et al*, 2018). Similarly, some SLE patients exhibit mutations in RNase H2 that increase rNMP levels in their genome (Günther *et al*, 2015). Mice carrying homozygous deletions of any of the RNase H2 subunits display embryonic lethality (Hiller *et al*, 2012; Reijns *et al*, 2012; Uehara *et al*, 2018). Human RNase H2 is likely also essential for embryonic development, as all mapped AGS mutations in RNase H2 subunits are missense mutations that still allow the production of full‐length proteins. The *in vitro* analysis of RNase H2 complexes harboring AGS mutations revealed that most of them preserve enzymatic activity with the exception of RNASEH2A‐G37S, a mutation located in the substrate recognition motif of the catalytic subunit (Rychlik *et al*, 2010). Additionally, the disease‐linked mutant proteins are not defective in assembling the heterotrimeric enzyme complex (Crow *et al*, 2006; Rohman *et al*, 2008; Kind *et al*, 2014), albeit complex stability is reduced for some mutations (Nishimura *et al*, 2019). The variation of retained RNase H2 function of AGS mutants was similarly observed in budding yeast (Potenski *et al*, 2019). The introduction of conserved AGS mutations in the yeast RNase H2 genes revealed defects in terms of genetic interactions, genome instability, and RER proficiency, which ranged between what could be observed for the wildtype enzyme and a full deletion (Potenski *et al*, 2019).

Solving the crystal structure of human RNase H2 in complex with an rNMP‐containing DNA substrate allowed to make predictions on the *in vivo* effect of specific point mutations (Figiel *et al*, 2011). The identified AGS mutations either map to the hydrophobic core or to the surface of RNase H2 and could therefore impact on substrate recognition or positioning, or may affect protein–protein interactions between the subunits or with other proteins. In support of the latter, *in vitro* data and imaging‐based *in vivo* approaches showed reduced complex stability and diminished recruitment of the mutated RNase H2 complex to sites of PCNA‐dependent DNA replication and repair (Kind *et al*, 2014). Other enzymes involved in nucleic acid metabolism have also been found to be mutated in AGS patients, including: the 3′ to 5′ single‐stranded DNA exonuclease TREX1 (Crow *et al*, 2006), the 3′ to 5′ exonuclease and dNTP hydrolase SAMHD1 (Rice *et al*, 2009), the RNA adenosine deaminase ADAR1 (Rice *et al*, 2012), and the cytosolic double‐stranded RNA receptor gene IFIH1 (Rice *et al*, 2014). RNase H2 mutations make up more than half of all reported AGS cases, followed by TREX1 (22%) and SAMHD1 (13%) mutations (Crow *et al*, 2015). Although different genes are affected, the overall disease pathology is very similar.

**Figure 5:**
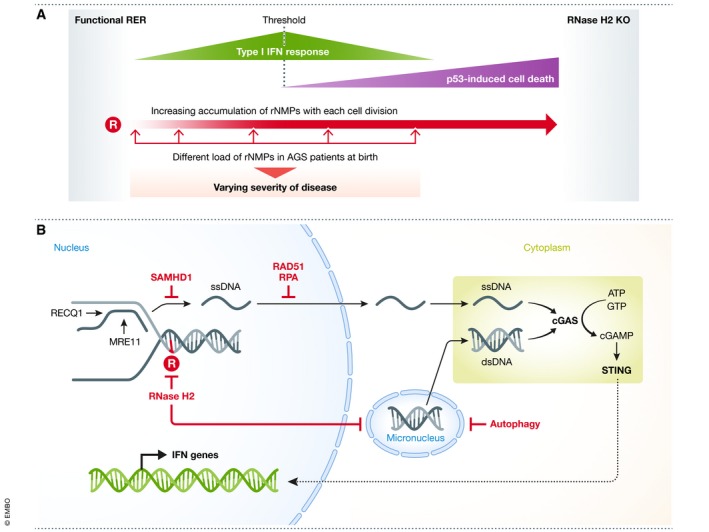
Impact of AGS mutations and IFN gene activation in disease (A) With increasing rNMP load in the genome, IFN‐regulated genes are upregulated. When rNMP levels cross a threshold, p53‐dependent cell death leads to a decreased IFN response but an enhanced phenotype. Different RNase H2 mutations identified in AGS patients could result in different initial rNMP levels at birth and explain the observed differences in disease severity between patients. (B) Release of nuclear DNA into the cytoplasm triggers activation of cGAS, which in turn activates STING that subsequently induces IFN genes. SAMHD1 prevents ssDNA release from stalled replication forks processed by RECQ1 and MRE11. RAD51 and RPA bind to and retain ssDNA in the nucleus. RNase H2 removes rNMPs (R in red circle) from DNA and thereby prevents formation of micronuclei. Autophagy can clear micronuclei, preventing them from rupture.

The severity of symptoms as well as the time of disease onset varies between patients (Rice *et al*, 2007). The majority of patients with RNase H2 mutations go through an initial phase of normal development before presenting an increased type I interferon (IFN) response indicative of upregulated immune signaling, followed by loss of neurological function and in most cases death before they reach adulthood. In patients with mutations in the RNASEH2B subunit, disease onset was significantly delayed, neurological function largely preserved, and patients displayed reduced mortality (Crow *et al*, 2015). This observed variation may be explained by the recent observation, based on expression of different RNase H2 variants in mice, that unrepaired rNMPs in the genome can be tolerated to some extent (Uehara *et al*, 2018). If rNMP levels remain below a certain threshold, they induce expression of genes typical of an innate immune response, but are permissive to embryonic development. Above this threshold, however, cells die in a p53‐dependent manner (Fig 5A). Considering that different mutations in RNase H2 could result in different levels of retained RER activity, the overall rNMP load could differ greatly between patients. In line with this, IFN levels measured in patients dropped with increasing age (Rice *et al*, 2007), accompanied by loss of mental and physical abilities. Considering the rNMP threshold model proposed by Uehara *et al*, the decrease of IFN signaling and the concomitant increase of disabilities observed by Rice *et al* could reflect the transition from IFN signaling to p53‐mediated cell death once the rNMP load crosses the threshold.

### Faulty RER leads to cGAS‐STING activation

Type I interferon signaling can be activated by the nucleic acid sensing cyclic GMP‐AMP synthase cGAS in conjunction with the adaptor protein STING. The cGAS‐STING axis elicits an innate immune response when the immune sensor cGAS recognizes free cytoplasmic DNA derived from pathogens, endogenous retroviral elements, mitochondrial DNA, or chromatinized nuclear DNA (Sun *et al*, 2013; Dhanwani *et al*, 2018). In addition, a type I interferon response can be activated by a nuclear fraction of cGAS that associates preferentially with centromeres (Gentili *et al*, 2019). In AGS, as revealed by work in patient‐derived cells and AGS mouse models, cytosolic cGAS‐STING mediates an upregulation of type I IFN genes and thus stimulates the autoimmune response (Mackenzie *et al*, 2016; Pokatayev *et al*, 2016). In RNase H2‐deficient cells, high levels of DNA damage (Hiller *et al*, 2012; Pizzi *et al*, 2015) increase the frequency of micronuclei, which have been found to colocalize with cGAS (Bartsch *et al*, 2017; MacKenzie *et al*, 2017). Upon breakdown of the unstable nuclear envelope encompassing a micronucleus, cGAS‐STING‐mediated recognition of the now cytoplasmic micronuclear DNA induces the expression of interferon‐induced genes (Fig 5B).

However, rNMP accumulation is likely not the only cause for cytoplasmic DNA species that trigger cGAS‐STING activity. AGS patients with SAMHD1 mutations do not display an accumulation of rNMPs (Lim *et al*, 2015), but the activation of an IFN response is similarly evoked by cGAS‐STING‐mediated sensing of endogenous DNA species in the cytoplasm (Coquel *et al*, 2018). SAMHD1 stimulates the exonuclease activity of MRE11 at stalled replication forks to facilitate ATR‐CHK1 activation and promote replication fork restart. In SAMHD1‐depleted cells, the RECQ1 helicase acts on stalled replication forks to displace ssDNA, which is then released by MRE11 and can become cytoplasmic (Coquel *et al*, 2018). Thus, altered processing of stalled forks can contribute to IFN induction. Since rNMPs lead to replication fork stalling and replication stress, it will be interesting to determine whether MRE11 also contributes to the accumulation of cytoplasmic DNA in the context of faulty RER (Fig 5B).

The involvement of cytoplasmic DNA in AGS etiology is further highlighted by the role of the exonuclease TREX1, another gene frequently mutated in AGS patients. TREX1 is located at the outer nuclear membrane, where it immediately degrades ssDNA that escapes into the cytoplasm (Wolf *et al*, 2016). The accumulation of ssDNA in the absence of TREX1 leads to an exhaustion of RAD51 and RPA, which in turn directly contributes to the generation of replication stress and p53‐dependent checkpoint signaling in addition to the activation of a cGAS‐STING‐mediated IFN response (Fig 5B). In line with this, depletion of RAD51 in both murine and human cell lines increases IFN signaling upon irradiating DNA damage (Bhattacharya *et al*, 2017). This suggests that the release of self‐derived DNA into the cytoplasm is a general phenomenon during DNA replication stress and is counteracted by mechanisms acting both at the stalled replication fork and in the cytoplasm. Characterizing in more detail how rNMPs interfere with DNA replication could allow for better understanding of how micronuclei and hence cytosolic nucleic acids arise, which eventually lead to autoimmunity in RNase H2‐mutated AGS patients.

Type I IFN signaling mediated by cGAS‐STING activates autophagy (Gui *et al*, 2019; Liu *et al*, 2019). At the same time, autophagy downregulates the IFN response by stimulating STING degradation (Prabakaran *et al*, 2018). Autophagy not only reduces IFN signaling in RNase H2‐deficient mouse embryonic fibroblasts (MEFs), but also clears accumulating micronuclei (Bartsch *et al*, 2017). Thus, inducing autophagy in AGS or SLE patients could offer a therapeutic approach based on two levels: (i) by downregulating IFN signaling through degradation of STING and dampening active autoimmunity in patients, and (ii) through clearance of immune‐stimulatory DNA species such as those contained in micronuclei (see Fig 5). Especially in early stages of disease, when autoimmune reactions are high and cell death has not yet occurred, patients might benefit from such therapy. Whether autophagy stimulation could prevent, or at least delay, the occurrence of the associated neurodegeneration and other symptoms attributed to cell death needs to be carefully evaluated. Recent work has demonstrated that replicative senescence due to telomere attrition induces cGAS‐STING activation and cell elimination through autophagy (Nassour *et al*, 2019). Therefore, although the stimulation of autophagy may lead to cGAS‐STING attenuation, it can also lead to enhanced cell elimination, which could exacerbate phenotypic onset, similar to what happens when p53 induces cell death if the rNMP threshold is reached (see above). Moreover, autophagy degrades nuclear lamina during cell senescence (Dou *et al*, 2015), thereby potentially exposing yet more endogenous DNA to cGAS. These concepts are still in their infancy, but require careful consideration in the context of AGS treatment.

The search for murine disease models of AGS has been challenging. Animals with full deletion of any RNase H2 subunit are inviable and die *in utero* unless p53 is co‐deleted (Hiller *et al*, 2012; Reijns *et al*, 2012). Cells from such embryos have enabled the characterization of DNA damage and genome instability phenotypes of AGS but could not give insight into the mechanism of IFN induction. Mechanistic dissection of IFN induction has instead been possible using expression of disease‐associated alleles in mice (Mackenzie *et al*, 2016; Pokatayev *et al*, 2016), but surprisingly, none of the mutant RNase H2‐expressing mice showed symptoms in their brains (Mackenzie *et al*, 2016), despite the fact that the brain is the organ most heavily affected in AGS and that patients display increased IFN activity in the cerebrospinal fluid (Crow *et al*, 2015). Brain‐specific conditional knockout of RNase H2 did not give rise to mice with a detectible brain phenotype either (Bartsch *et al*, 2018). Bartsch *et al* argue that a mouse brain undergoes fewer cell divisions than the human brain and might thus not reach the load of genomic rNMPs required to induce neuropathology (Uehara *et al*, 2018). In support of this notion, isolated astrocytes from those mouse brains recapitulated defects associated with AGS when cultured under mitogenic conditions, exhibiting high levels of DNA damage, increased type I IFN signaling, and limited proliferative capacity (Bartsch *et al*, 2018). As transgenic mice that overexpress type I IFN specifically in the brain display signs of neuropathology (Akwa *et al*, 1998), astrocytes from RNase H2‐deficient mouse brains could be a valuable tool to understand the transition between IFN signaling and the induction of cell death in AGS.

## RER intermediates can also contribute to neurological disease

Even in the presence of a functional RER pathway, genomic rNMPs can still contribute to the development of another neurological disease, ataxia with oculomotor apraxia 1 (AOA1). This neurological syndrome is caused by mutations in APTX, the gene encoding the aprataxin enzyme (Moreira *et al*, 2001). Aprataxin promotes the repair of adenylated DNA ends (both at double‐strand breaks and at DNA nicks) that can arise due to abortive DNA ligation, but also upon premature ligation of nicked abasic sites (Ahel *et al*, 2006; Rass *et al*, 2007). By removing the covalently bound 5′‐adenosine monophosphate (5′AMP), aprataxin creates DNA ends that can be processed by DNA ligases. Structural studies of aprataxin proteins carrying mutations found in AOA1 patients suggest that the majority of mutations affect protein stability to various degrees, whereas one mutation directly interferes with the deadenylation reaction, and another variant causes allosteric changes of the active site conformation (Tumbale *et al*, 2018).

A nick flanked by a 3′OH and a 5′phosphate with a single rNMP located at its 5′‐side, a structure that would typically be created by RNase H2‐mediated incision (discussed above), is less‐efficiently ligated *in vitro* than nicks at dNMPs (Tumbale *et al*, 2014). Due to this inefficient ligation, the exposed 5′phosphate can become adenylated to yield a non‐repairable 5′AMP product. Without rapid processing, the RNase H2 action on rNMPs therefore creates substrates (5′AMP) for aprataxin. When aprataxin is mutated, the accumulation of irreparable 5′AMP consequently contributes to disease pathology in AOA1 patients. In line with this, *S. cerevisiae* cells lacking the aprataxin homolog Hnt3 are inviable when the incorporation of rNMPs is increased using a DNA Pol ε mutant (*pol2‐M644G*), but can be rescued by deletion of *RNH201* (Tumbale *et al*, 2014).

## RNase H2 mutations are implicated in cancer

**Figure 6:**
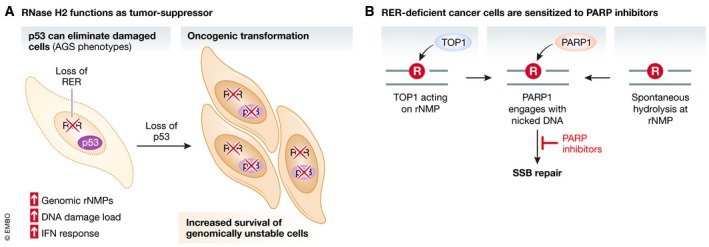
Tumor suppressor functions of RNase H2 and RER (A) Loss of RER leads to increased genomic rNMP accumulation and the indicated consequences, but damaged cells are eliminated as long as p53 is present. In the nervous system, cell elimination likely leads to the neurodegenerative phenotypes associated with AGS. Genome instability upon p53 loss can lead to oncogenic rearrangements and cancer development. (B) rNMPs that accumulate in the absence of RER are either cleaved by TOP1 or hydrolyzed spontaneously, creating single‐strand breaks (SSB). These nicks recruit PARP, sensitizing cells to PARP inhibitors that will create toxic PARP‐trapping lesions.

Cells with dysfunctional RER experience high loads of DNA damage. Recent studies imply RER as an important tumor suppressor mechanism. When RNase H2 is disrupted in murine epidermal cells, a tissue with rapid cell turnover, type I IFN signaling and spontaneous DNA damage increase in the skin (Hiller *et al*, 2018). All RNase H2 epithelial knockout mice analyzed in this study developed squamous cell carcinoma (SCC) or precursor forms of skin cancer within less than a year. Concomitant loss of p53 led to enhanced skin inflammation and survival of more damaged cells, suggesting that the p53 DNA damage response limits oncogenic transformation of RER‐deficient epidermis through the elimination of highly damaged cells (Fig 6A).

Similarly, deletion of RER in murine intestinal epithelial tissue, especially in the absence of p53, increased the development of intestinal and colorectal tumors (Aden *et al*, 2019). A potential link between non‐functional RER and human cancer is supported by analyses of publicly available transcriptomic (RNA‐seq) datasets from colorectal cancer (CRC) patients. Aden *et al* (2019) describe a correlation of decreased RNase H2 expression and poor survival rates, the importance of which remains to be evaluated.

A more direct analysis of the interconnection of cancer and defective RER in humans is difficult, as most AGS patients with hereditary RNase H2 mutations have a short lifespan (Crow *et al*, 2015). However, loss of RNase H2 function has been reported in chronic lymphocytic leukemia (CLL) and metastatic castration‐resistant prostate cancer (Zimmermann *et al*, 2018). Determination of RNASEH2B copy numbers in 100 patient‐derived CLL cell lines and 226 prostate cancers showed that at least one copy was lost in 57% and 36% of the respective cancer samples. Two loci on chromosome 13q14 frequently lost in those cancer types (microRNA cluster *DLEU2‐mir‐15‐16* in CLL, and *RB1* locus in prostate cancer) are located in close proximity to the RNASEH2B gene. Consequently, RNASEH2B is simultaneously lost upon deletion of these tumor suppressors.

The determination of RNASEH2B copy numbers in human cancer can become significant in the context of therapy, because RER‐deficient cells are sensitive to (poly‐ADP)ribose polymerase (PARP) inhibition. Mouse xenograft studies confirmed that tumors arising from implanted RER‐deficient CLL cells are similarly sensitized. Mechanistically, cell death is likely caused by PARP1‐trapping at DNA lesions that are created by TOP1‐mediated repair attempts at rNMPs (Zimmermann *et al*, 2018; Fig 6B). However, substrates for PARP1 could also arise in a TOP1‐independent manner through spontaneous hydrolysis of DNA at unrepaired rNMPs. This is supported by the notion that PARP1 can sense unligated Okazaki fragments due to the presence of DNA nicks between them, and signal for their repair (Hanzlikova *et al*, 2018). The loss of RNase H2 activity further renders cells hypersensitive to ATR inhibition, which is likely linked to replication stress when rNMPs are encountered by the replisome (Wang *et al*, 2018; Hustedt *et al*, 2019).

A detailed analysis of existing human cancer genomes could reveal other types of cancer that coincide with a loss of RNase H2 function. This is particularly important given the recent findings with regard to RNase H2 status and sensitivity to PARP or ATR inhibitors, as it may have important consequences with respect to therapeutic approaches in patients (Wang *et al*, 2018; Zimmermann *et al*, 2018; Hustedt *et al*, 2019). Therefore, it seems that the status of the p53 and/or pRb checkpoints may be critical in the fate of RER‐defective cells. Whereas p53‐mediated cell death appears to anticipate loss of neuronal function in AGS, it can nonetheless act as a tumor suppressor.

Finally, RNase H2 may be involved in cancer development and progression via additional, so far uncharacterized functions. One recent report identified reduced expression specifically of the RNASEH2C subunit, independent of enzymatic activity and not associated with an induction of the DNA damage response, as a driver of breast cancer metastasis in mice (Deasy *et al*, 2019). These authors also did not observe activation of cGAS‐STING signaling, but instead increased cytotoxic T‐cell‐mediated immune responses, highlighting the need to characterize the cancer roles of RNase H2 in more detail.

## Conclusions

Ribonucleotide insertions into the genome cannot be avoided during DNA replication. How and when they are dealt with, however, can have significant consequences with respect to mutation frequency, genome stability, cell viability, and disease. In the best‐case scenario, RNase H2 excises the rNMP via the canonical RER pathway to achieve error‐free repair (Fig 1). New insights have illustrated the importance of limiting this reaction to a postreplicative phase to ensure that DNA nicking activity does not occur at the same time as DNA replication. How this temporal regulation is achieved still remains to be elucidated, as do many other questions with regard to how RER is achieved *in vivo*, including the relevance and nature of the interaction between RNase H2 and the replisome through PCNA (Fig 3).

When RER is impaired, there are two immediate consequences: (i) rNMPs accumulate to increased levels and (ii) Top1 takes over and removes rNMPs, but at a costly price to the integrity of the genome. The consequences of rNMP accumulation remain poorly described, and here, we have attempted to summarize the potential effects on chromatin state, transcriptome and RNAP II status and DNA repair by summarizing what is known from the literature (Fig 4). The Top1‐induced rNMP removal pathway, on the other hand, has been more extensively investigated and is associated with the induction of DNA damage and loss of genomic integrity (Fig 2). In AGS autoimmune disease, where neuronal RNase H2 function is compromised, genome instability can lead to the formation of micronuclei and activation of cGAS/STING, which then triggers an inflammatory response. The eventual activation of p53 will lead to cell elimination, which is responsible for the AGS phenotypes (Fig 5). In the absence of a checkpoint response, the mutator phenotype may drive oncogenic transformation (Fig 6). Indeed, loss of RNase H2 has recently been linked to different types of human cancer.

Due to the frequency of faulty rNTP insertions into the genome, RER defects lead to severe human pathologies. Nonetheless, our increased mechanistic understanding of this pathway now paves the way for possible new therapeutic strategies. In this respect, it is important to continue to understand RER and its regulations as well as the subsequent consequences when RER fails.

## Conflict of interest

The authors declare that they have no conflict of interest.
